# Utilizing deep learning models in an intelligent eye-tracking system for autism spectrum disorder diagnosis

**DOI:** 10.3389/fmed.2024.1436646

**Published:** 2024-07-19

**Authors:** Nizar Alsharif, Mosleh Hmoud Al-Adhaileh, Mohammed Al-Yaari, Nesren Farhah, Zafar Iqbal Khan

**Affiliations:** ^1^King Salman Center for Disability Research, Riyadh, Saudi Arabia; ^2^Department of Computer Engineering and Science, Albaha University, Al Bahah, Saudi Arabia; ^3^Deanship of E-learning and Information Technology, King Faisal University, Al-Ahsa, Saudi Arabia; ^4^Department of Chemical Engineering, College of Engineering, King Faisal University, Al-Ahsa, Saudi Arabia; ^5^Department of Health Informatics, College of Health Sciences, Saudi Electronic University, Riyadh, Saudi Arabia; ^6^Department of Computer Science, College of Computer and Information Sciences, Prince Sultan University, Riyadh, Saudi Arabia

**Keywords:** autism spectrum disorder, eye tracking, deep leaning, VGG19, MobileNet, DenseNet169, hybrid model

## Abstract

Timely and unbiased evaluation of Autism Spectrum Disorder (ASD) is essential for providing lasting benefits to affected individuals. However, conventional ASD assessment heavily relies on subjective criteria, lacking objectivity. Recent advancements propose the integration of modern processes, including artificial intelligence-based eye-tracking technology, for early ASD assessment. Nonetheless, the current diagnostic procedures for ASD often involve specialized investigations that are both time-consuming and costly, heavily reliant on the proficiency of specialists and employed techniques. To address the pressing need for prompt, efficient, and precise ASD diagnosis, an exploration of sophisticated intelligent techniques capable of automating disease categorization was presented. This study has utilized a freely accessible dataset comprising 547 eye-tracking systems that can be used to scan pathways obtained from 328 characteristically emerging children and 219 children with autism. To counter overfitting, state-of-the-art image resampling approaches to expand the training dataset were employed. Leveraging deep learning algorithms, specifically MobileNet, VGG19, DenseNet169, and a hybrid of MobileNet-VGG19, automated classifiers, that hold promise for enhancing diagnostic precision and effectiveness, was developed. The MobileNet model demonstrated superior performance compared to existing systems, achieving an impressive accuracy of 100%, while the VGG19 model achieved 92% accuracy. These findings demonstrate the potential of eye-tracking data to aid physicians in efficiently and accurately screening for autism. Moreover, the reported results suggest that deep learning approaches outperform existing event detection algorithms, achieving a similar level of accuracy as manual coding. Users and healthcare professionals can utilize these classifiers to enhance the accuracy rate of ASD diagnosis. The development of these automated classifiers based on deep learning algorithms holds promise for enhancing the diagnostic precision and effectiveness of ASD assessment, addressing the pressing need for prompt, efficient, and precise ASD diagnosis.

## 1 Introduction

Autism Spectrum Disorder (ASD) is a neurological condition that involves complications in both spoken and non-spoken communication, as well as challenges in social interaction. It is also marked by monotonous and stereotyped behaviors (1). The intensity of indicators and the impact of ASD differ from one circumstance to another. As to the Centers for Disease Control and Prevention (CDC), the commonness of ASD is assessed to be 1 in 54 children. This condition affects individuals from diverse racial, ethnic, and socioeconomic backgrounds. Furthermore, the prevalence of ASD in boys is four times higher than in girls. Additionally, girls with ASD often have fewer observable symptoms compared to boys (2). Autism is a persistent and enduring condition that remains present throughout a person’s whole life (3). Hence, it is of utmost importance to identify ASD at an early stage, since individuals who are identified with ASD during early infancy can greatly benefit from suitable therapies, leading to a favorable long-term result (4).

Facial expressions communicate a wealth of personal, emotional, and social information from early infancy. Even in a short interaction, people may effortlessly focus on and rapidly comprehend the intricate details of a person’s face, accurately identifying their emotional state and social situation, and frequently recalling their face later (5). Neuroimaging research has indicated that eye interaction can stimulate brain movement in parts of the brain associated with social interactions. Additionally, studies on human development have provided evidence that infants and young children have a natural inclination to pay attention to and comprehend faces that make direct eye contact. Increasing evidence suggests that ASD is related with an aberrant design pattern of eye tracking conduct (6, 7). Therefore, it is widely accepted that autism is characterized by impairments in facial handling. Nevertheless, the precise attributes of these discrepancies and the correlations among atypical face processing and deviant socio-emotional function in ASD remain inadequately comprehended.

Eye tracking, a non-invasive and straightforward measurement technique, has garnered the attention of scientists in recent years (8–11). The use of eye tracking in ASD research is justified by the correlation between ASD and different attention patterns, which differ from those seen in typical development (12–15). Hence, the use of eye tracking based system to quantify eye activities and gaze designs should assist in understanding the aberrant behavior associated with persons diagnosed with ASD, as well as distinguishing individuals with ASD from typically developing (TD) individuals. Eye tracking is a method used by certain computational systems to aid in the identification of mental problems (16, 17). Eye tracking technology is beneficial in addressing ASD, a neurodevelopmental disease marked by challenges in social communication and repetitive activities. An early indication of ASD is the absence of visual engagement, namely the lack of eye contact. This trait is seen in infants as early as six months old, irrespective of the cultural context in which they are raised. Eye-tracking technology is essential in diagnosing ASD through the analysis of visual patterns (18). A device based on eye-tracking framework classically comprises a high-determination digital camera device and a sophisticated technique based machine learning algorithm that accurately determines the coordinates of eye gaze when persons watch films or pictures. This technology’s eye gaze data may help customize therapy to ASD patients’ social issues (19). To further understand how eye-tracking biomarkers might discriminate ASD subgroups, we should explore the effects of closely related mental illnesses such as attention deficit hyperactivity disorder (ADHD), nervousness, and attitude complaint. We may better understand how these variables may affect our ability to distinguish different groups in a medical setting by doing this. Research indicates that children who having the cases of Autism ASD and ADHD tend to have shorter periods of focused attention on faces while looking at static social cues that are not very complex, compared to children who simply have ASD and those with TD (20).

Research has shown that eye-tracking data can be utilized as medical indicators that can be applied in medical health domain to identify ASD in children at an initial state (18). Biomarkers, sometimes referred to as biological markers, are quantifiable and impartial signs that offer insights around a patient’s apparent organic state. Bodily fluids or soft tissue biopsies are frequently employed to assess the efficacy of handling for a disease or medicinal disorder.

A crucial element of social interaction is maintaining eye contact, a skill that individuals with ASD often find challenging. Eye tracking technology may be applied to measure the length of time someone maintains eye interaction and the occurrence and track of their eye movements. This provides measurable signs of difficulties in social interactions. Individuals with ASD may also exhibit other irregularities in pictorial processing, including heightened focus on specific details, sensory hypersensitivity, and difficulties with complex visual tasks. Hence, the sophisticated deep learning algorithms, namely MobileNet, VGG19, DenseNet169, and the hybrid of MobileNet-VGG19, were applied for the early-stage recognition of ASD. The primary contributions of this research article are as follows:

- This work introduces a new method for creating eye-tracking event detectors using a deep learning methodology.
- The research asserts that it has attained accuracy (100%) in identifying ASD by employing the MobileNet algorithm. This indicates that the DenseNet169 and hybrid of MobileNet-VGG19 model that was created has demonstrated encouraging outcomes in accurately differentiating persons with ASD from those who do not have ASD, using eye tracking data.
- The proposed methodology was compared with different existing systems that used the same dataset; it is observed that our model achieved high accuracy because we have used a different preprocessing approach from improving dataset.
- This work presents an innovative artificial intelligence (AI) technique for the diagnosis of ASD. Its objective is to differentiate persons with autism from those without utilizing deep learning models, relying on publicly accessible eye-tracking datasets. The suggested approach was evaluated against other existing systems that utilized the same dataset. It was found that the proposed system achieved a high accuracy rate of 100% when compared to one of the deep learning models.

## 2 Background

ASD can be detected by early screening techniques utilizing DL algorithms. These approaches have become more prominent because of their accuracy rate and capability to grip large volumes of data. It assists experts in automating the diagnostic procedure and reducing the time spent on tests (21, 22). AI techniques are used in the rehabilitation process to lessen symptoms of ASD. This research analyzes the utilization of DL approaches in the past five years for diagnosing ASD through the application of eye tracking techniques.

Fang et al. (23) introduced a novel method for identifying children with ASD based on stimuli that include the ability to follow someone’s gaze. Individuals with ASD exhibited typical patterns of visual attention, especially while observing social settings. The scientists developed a novel deep neural network (DNN) method to abstract distinctive characteristics and categorize children with ASD and healthy controls based on individual images.

Elbattah et al. (24) developed a machine learning (ML)-based approach to aid in the diagnosing process. This approach relies on acquiring knowledge of sequence-oriented patterns in action eye motions. The primary philosophy was to represent eye-tracking data as written documents that analyze a sequence of rapid eye movements (saccades) and periods of gaze fixation. Therefore, the study utilized the natural language processing (NLP) technique to transform the unorganized eye-tracking information.

Li et al. (25) introduced an automated evaluation framework for detecting typical intonation patterns and predictable unique phrases that are important to ASD. Their focus was on the linguistic and communication difficulties experienced by young children with ASD. At first, the scientists utilized the Open SMILE toolkit to extract high-dimensional auditory characteristics at the sound level. They also employed a support vector machine (SVM) backend as the standard baseline. Furthermore, the researchers suggested many DNN arrangements and structures for representing a shared prosody label derived directly from the audio spectrogram after the constant Q transform.

Identification and intervention for ASD have enduring effects on both ASD children as well as their families, necessitating informative, medical, social, and economic assistance to enhance their overall well-being. Professionals have problems in conducting ASD assessments due to the absence of recognized biophysiological diagnostic techniques (25, 26). Therefore, the diagnosis is often determined by a thorough evaluation of behavior, using reliable and valid standardized techniques such as the Autism Diagnostic Observation Schedule (ADOS) (27) and the Autism Diagnostic Interview-Revised (ADI-R) (28). These tools, widely approved in investigation and research domains, are considered the most reliable method for diagnosing ASD in medical situations (29, 30). However, using them involves the use of many materials, a significant amount of time, and is somewhat expensive (25, 26). Furthermore, the diagnostic technique necessitates the involvement of skilled and knowledgeable interviewers, who have the potential to influence the process. This is accompanied by the inclusion of intricate clinical procedures (25, 31). Collectively, these difficulties frequently contribute to a postponed identification, leading to a delay in the initiation of early intervention (26). Research indicates that early treatments for children with ASD before the age of five result in a much higher success rate of 67%, compared to a success rate of just 11% when interventions begin after the age of 5 (32).

Eye-tracking technology is regarded as a beneficial method for doing research on ASD since it allows for the early detection of autism and its characteristics (33, 34) in a more objective and dependable manner compared to traditional assessments (35). There has been a significant rise in the amount of eye-tracking research focused on autism in the past period. This increase can be attributed to improved accessibility to eye-tracking technology and the development of specialized devices and software that make recording eye-tracking data easier and more cost-effective.

Machine learning and eye-tracking devices are often used together. Data-driven machine learning uses sophisticated mathematics learning, statistical estimates, and information theories (36, 37). This method trains a computer program to examine data and find statistical trends (36–39). Machine learning may improve autism investigation studies by giving an unbiased and reproduceable second evaluation (18), including initial autism detection (40), analysis (41), behavior (16), and brain activity (17). Machine learning may also be a viable biomarker-based tool for objective ASD diagnosis (42). ASD is diagnosed via machine learning in IoT systems (43). By helping ASD youngsters learn, assistive technology may improve their lives. This method is backed by studies (44).

Various studies have utilized artificial neural network (ANN) to classify cases of ASD. For example, in ref. (18), the authors investigated the integration of eye-tracking technologies with ANN to assist in the detection of ASD. Initially, other approaches that did not use neural networks were used. The precision achieved by this ensemble of models was adequate. Subsequently, the model underwent testing using several ANN structures. According to the results, the model with a single layer of 200 neurons achieves the maximum level of accuracy. In ref. (45), researchers examined ASD children’s visual attention when observing human faces. They extract semantic characteristics using DNN. When viewing human faces, ASD feature maps differ from those without ASD. These feature maps are combined with CASNet features. They contrasted CASNet to six different deep learning based techniques. CASNet has outdone all other models in every situation. The scientists used eye movement patterns to classify children with TD and ASD (46). They combined CNNs with LSTMs. CNN-LSTM extracted features from saliency maps and scan route fixation points. SalGAN pretrained prediction model preprocessed and input network data. The validation dataset accuracy of the proposed model is 74.22%.

Akter et al. (47) proposed a method that uses transfer learning to identify ASD by analyzing face features. They developed an improved facial recognition system using transfer learning, which can accurately identify individuals with ASD.

Raj and Masood (48) utilized several machine and deep learning techniques with the aim of identifying ASD in youngsters. They utilized three publicly available datasets obtained from the UCI Repository.

Xie et al. (49) proposed a two-stream deep learning network for the detection of visual attention in individuals with ASD. The suggested framework was built using two VGGNets that were derived from the VGG16 architecture and were similar to each other.

## 3 Methods

**Figure 1:**
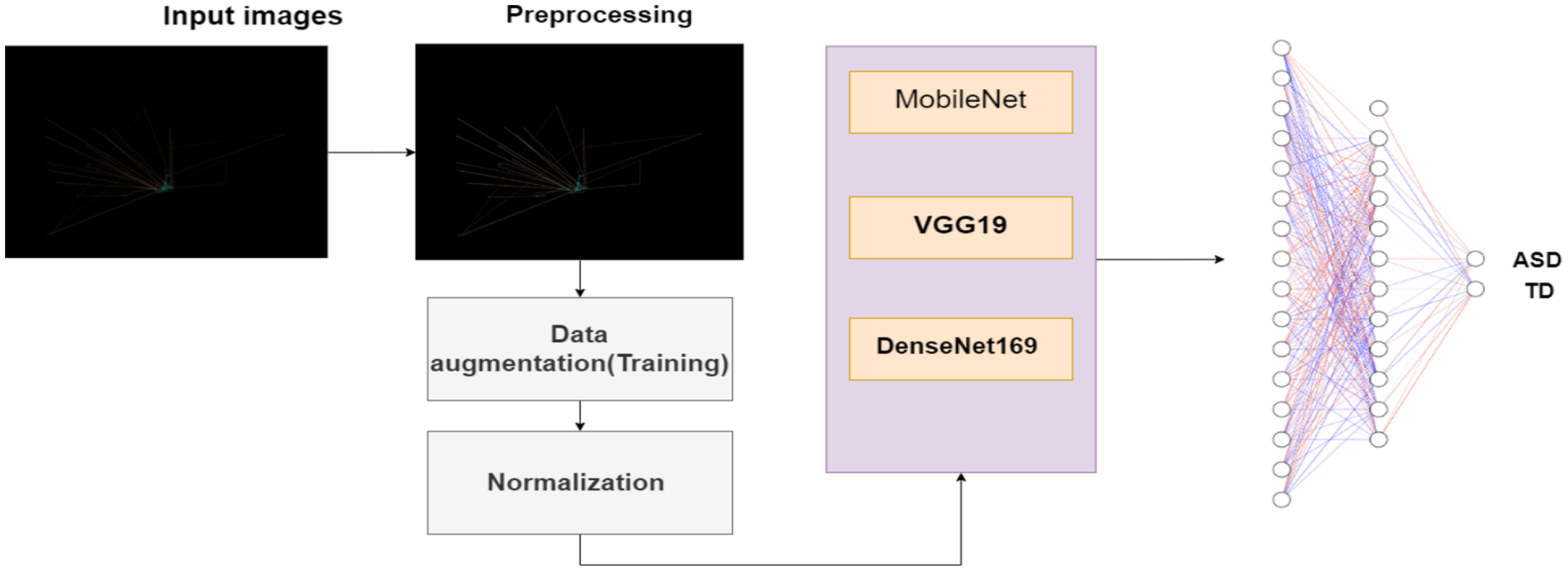
Structure of the proposed methodology.

This section presents in depth the planned methodology applied to develop ASD detection system using deep learning techniques capable to detect ASD from eye tracking images based features. This methodology includes dataset collection, data preprocessing, deep learning classification model, evaluation metrics and results analysis. The framework of this methodology is shown in Figure 1.

### 3.1 Dataset

**Figure 2:**
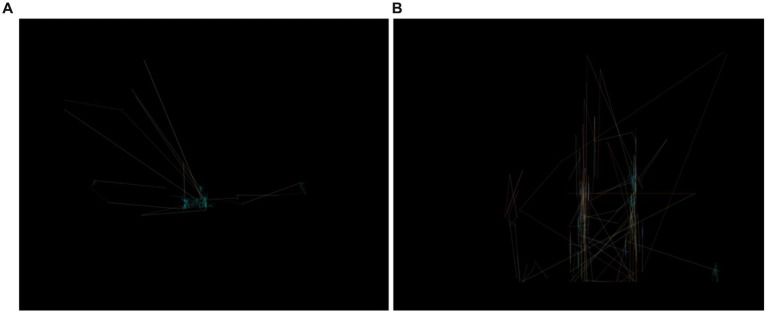
Sample of images: **(A)** ASD **(B)** TD.

The dataset was obtained from a public repository that contains eye-tracking images. The collection presently comprises 547 images. The default images dimensions were established at 640 × 480. More precisely, there were 328 images for the people without ASD, and 219 images for the persons diagnosed with ASD. Figure 2 shows samples of eye-tracking images that were used for examining the proposed methodology.

### 3.2 Data preprocessing

It is an important step in making the images dataset for training machine learning models. We applied various data preprocessing methods to make certain the dataset is suitable for model training which are discussed as follows.

- *Image Resize:* The first step in data preprocessing encompasses resizing all images in the dataset to a standard size of 640 × 480 pixels. This ensures uniformity in image measurements and facilitates effective processing during model training.
- *Image Enhancement*: For all images in the dataset used, we applied a specific preprocessing step by improving their resolution by 20% using the Image Enhance module. This enhancement aims to enhance the quality and clarity of the images data, particularly for those where it’s considered necessary.
- *Vectorization:* After resizing and enhancing the images, we converted them into numerical arrays using vectorization techniques. This step includes transforming each image into a multi-dimensional array of pixel values, making it compatible with computational operations and deep learning algorithms.
- *Normalization:* after transformation to numerical arrays, we normalized the pixel values to fall within the range of [0, 1]. Normalization ensures that the pixel values are scaled appropriately, facilitating more stable and efficient model training by preventing issues related to large variations in input images data.
- *Splitting Data*: Once the images are preprocessed and converted into numerical arrays, we divide the dataset into three sets namely training, validation, and testing. This step is essential for evaluating model results, as it allows us to train the model on one subset of data, validate its performance on another subset, and finally test its generalization ability on a separate unseen subset.
- *Data Augmentation*: To increase the diversity and robustness of the training dataset, data augmentation techniques, using the Image Data Generator module, was applied. This method involve rotation, shifting, and flipping of images, introducing variations that help avoid overfitting and enhance the model’s capability to be generalized to new, unseen images data.

### 3.3 Improving the deep leaning algorithms

#### 3.3.1 The VGG19 model

**Figure 3:**
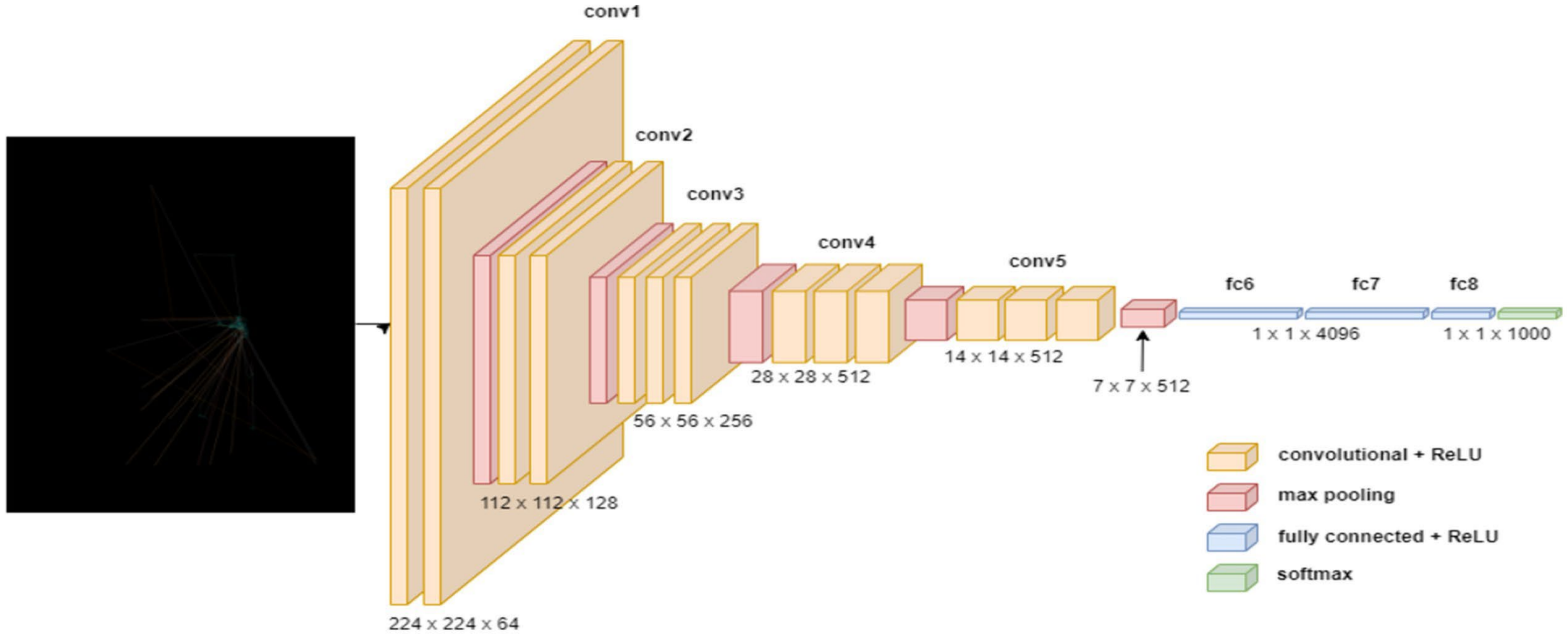
Structure of the VGG19 model.

The VGG19 model (50) is a sequential model architecture constructed in this study for the purpose of detecting ASD based on eye-tracking features. Initially, the model incorporates the pre-trained VGG19 architecture, with the weights initialized from the ImageNet dataset, excluding the fully connected layers, and specifying the input shape to match the dimensions of the input images with size of (640, 480). Subsequently, a GlobalAveragePooling2D layer is added to obtain a condensed representation of the features extracted by VGG19. Following this, several dense layers are appended to the model, comprising 1,024, 128, and 64 neurons, each activated by the rectified linear unit (ReLU) function, to facilitate the learning of intricate patterns within the data. Lastly, a Dense layer with 2 units and a softmax activation function are employed for binary classification, enabling the model to predict the probability of ASD presence. Figure 3 shows the VGG1 model structure.

Upon compiling the model, utilizing the sparse categorical cross-entropy loss function and RMSprop optimizer with a learning rate of 0.0001, data augmentation approach is adopted throughout training process to improve the model’s generality competences. Through this architecture, the model aims to effectively discern the presence of ASD based on the provided eye-tracking features, leveraging the robustness of the VGG19 convolutional neural network. Table 1 outlines the parameters of VGG19 model.

**Table 1:** Parameters of the VGG19 model.

Parameter	Description
Architecture	Sequential
Base model	VGG19 (pre-trained on ImageNet)
Input shape	(640, 480, 3)
Global pooling layer	Global Average Pooling 2D
Dense layers	1,024, 128, 64 neurons with ReLU activation
Output layer	Dense layer with 2 units, softmax activation (binary classification)
Loss function	Sparse categorical cross-entropy
Optimizer	RMSprop with learning rate of 0.0001
Metrics	Accuracy
Data augmentation	Applied during training using Image Data Generator
Training batch size	16
Validation batch size	32
Number of epochs	100

#### 3.3.2 The MobileNet model

The MobileNet (51) model architecture has a sequential model structure, which allows for the systematic building of a neural network layer by layer. The MobileNet pre-trained convolutional neural network (CNN) is used as the basis model in this methodology, which is prepared with learnt representations from the ImageNet dataset. However, the fully connected layers of the MobileNet are excluded to facilitate transfer learning. Following integration of the MobileNet base model, a Global Average Pooling 2D layer is used to compress the three-dimensional spaces of the feature maps formed by the convolutional layers. The pooling layer calculates the mean value of each feature map over all spatial locations, resulting in a fixed-size vector representation of the input image, regardless of its size.

Successively, many dense (completely linked) layers are added to capture more complex characteristics and perform classification tasks. The dense layers are composed of 1,024, 128, and 64 neurons, respectively, each of which is activated using the ReLU activation function. The ReLU activation function is selected for its capacity to introduce non-linearity, hence improving the complexity of the model and the efficiency of training.

**Figure 4:**
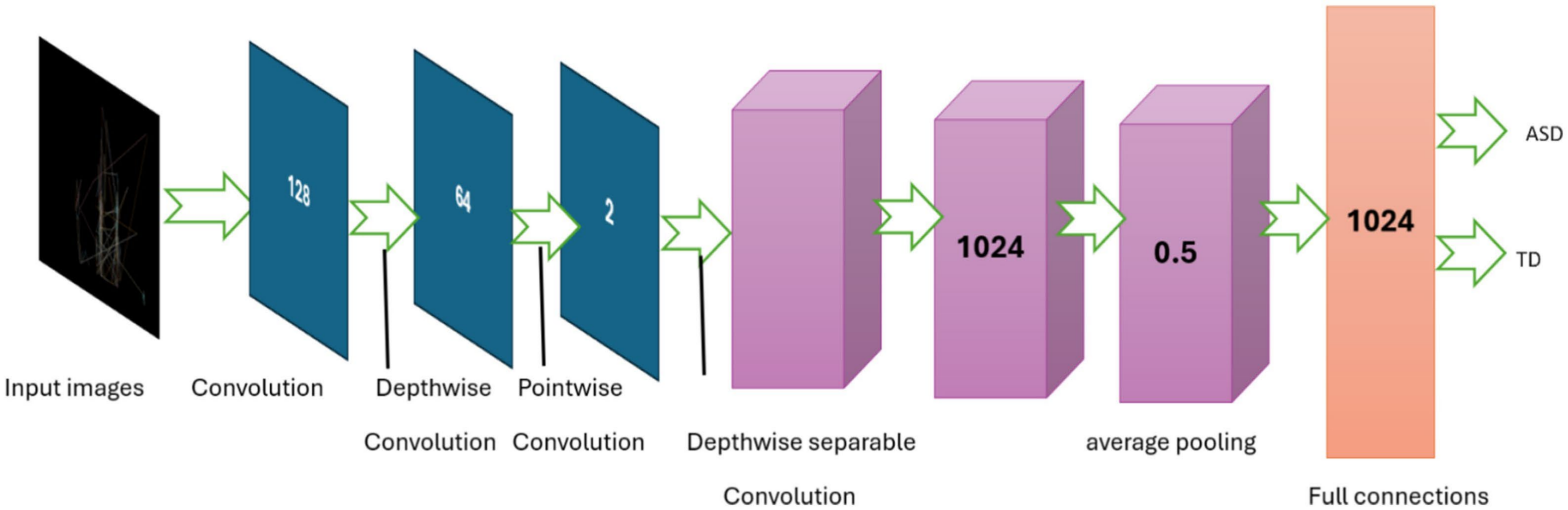
Structure of the MobileNet model.

The classification layer of the model that is named as output layer consists of a dense layer with 2 units, representing the two classes for binary classification (ASD or TD). These units are activated using the softmax function. This function generates probability for every class. This model architecture seeks to utilize the data obtained by MobileNet and conduct classification based on these features. It then proceeds to fine-tune the dense layers to suit the particular purpose of ASD detection using eye-tracking features. The MobileNet architecture is presented in Figure 4 and model’s parameters are listed in Table 2.

**Table 2:** Parameters of the MobileNet model.

Parameter	Description
Architecture	Sequential
Weights	Image net
Input shape	(640, 480, 3)
Pooling layer	0.5
Dense	256
Output layer	Dense layer with 2 units, softmax activation (binary classification)
Loss function	Sparse categorical cross-entropy
Optimizer	adam with learning rate of 0.0001
Metrics	Accuracy
Data augmentation	Applied during training using Image Data Generator
Training batch size	16
Validation batch size	32
Number of epochs	100

#### 3.3.3 The DenseNet169 model

**Figure 5:**
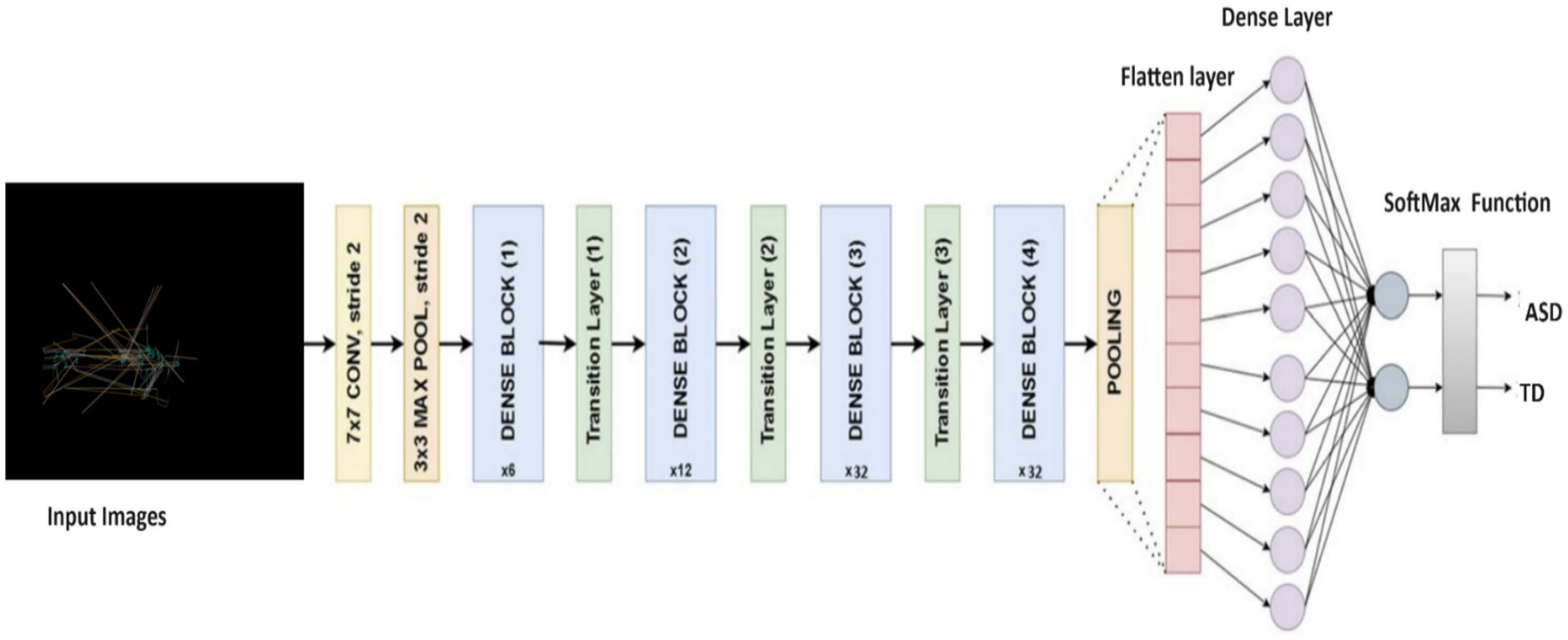
Structure of the DenseNet 169 model.

We also applied the DenseNet169 (52) model as the base, which is tailored for ASD detection based on eye-tracking features. Utilizing pre-trained weights from the ImageNet dataset, the model excludes the fully connected layers for transferring learning tasks. After integrating a Global Average Pooling 2D layer to condense feature maps, dense layers capture higher-level features. Dropout layers mitigate overfitting, and the output layer, activated by softmax, produces class probabilities. With frozen base model layers, the model is compiled with appropriate functions and benefits from learning rate scheduling. Data augmentation enhances training, aligning with the ASD detection task’s needs. Figure 5 displays the structure of DenseNet169 model, and Table 3 outlines the parameters used in DenseNet169 model.

**Table 3:** Parameters of the DenseNet169 model.

Parameter	Description
Base model	DenseNet169 pre-trained CNN initialized with ImageNet weights, excluding fully connected layers
Global Average Pooling 2D	Condenses spatial dimensions of feature maps
Dense layers	512 and 256 neurons with ReLU activation, capturing higher-level features
Dropout layers	Dropout rate of 0.5 for regularization, preventing overfitting
Output layer	Dense layer with 2 units for binary classification, activated by softmax
Frozen base model layers	Retains learned features during training
Loss function	Sparse categorical cross-entropy
Optimizer	RMSprop with learning rate of 0.0001
Learning rate scheduler	Reduces learning rate based on validation loss
Data augmentation	Applied during training to improve generalization

#### 3.3.4 The hybrid model

**Figure 6:**
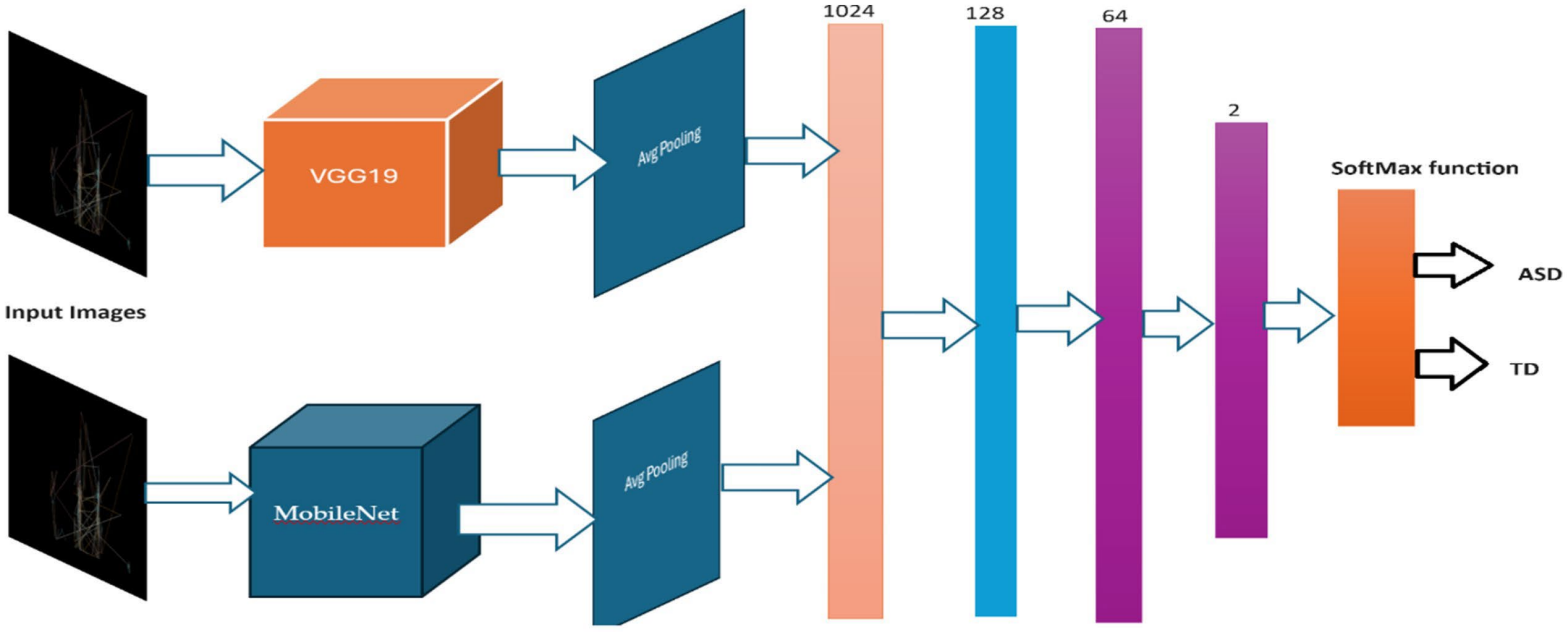
Structure of the hybrid model of VGG19 and MobileNet.

The framework of this a combination model employs the capacities of two solidified convolutional neural network (CNN) structures, VGG19 (46) and MobileNet (51) models, to enhance its efficacy in recognizing ASD using eye-tracking features. At first, the model provides in the pre-trained VGG19 and MobileNet structures, although without their completely connected layers. It then freezes all layers to maintain their learnt representations. Global Average Pooling 2D layers are subsequently employed to acquire feature representations from the output of each model. These representations are merged to develop a united feature vector, which is then handled through numerous robust layers to capture complicated data patterns. Following that, the model is collected utilizing acceptable loss and optimization functions, while data augmentation approaches are employed during training to improve its generalization capability. This hybrid model aims to improve classification accuracy in the ASD detection task by combining the features learned by VGG19 and MobileNet. By using the capabilities of both architectures, it seeks to attain heightened accuracy. Table 4 summarizes the parameters used in the hybrid VGG19-MobileNet model, and Figure 6 displays the structure of hybrid model.

**Table 4:** Parameters of the hybrid model.

Parameter	Description
Pre-trained models	VGG19 and MobileNet are used as pre-trained CNN architectures.
Trainable layers	All layers in both VGG19 and MobileNet models are frozen
Output layers	Global Average Pooling 2D layers are added to the output of each model
Concatenated output	The outputs of both models are concatenated to create a fused feature vector
Dense layers	Several dense layers with ReLU activation functions: 1024, 128, and 64 units
Output activation	Softmax activation function is used for the output layer
Loss function	Sparse categorical cross entropy loss function is used
Optimizer	RMSprop optimizer with a learning rate of 0.0001 is employed
Data augmentation	Image data augmentation techniques are applied during training
Training epochs	The model is trained for 100 epochs
Batch size	Batch size is set to 16 for training and 32 for validation

### 3.4 Evaluation metrics

Assessing the performance and testing results obtained by the proposed deep learning models namely MobileNet, VGG19, DenseNet169 and hybrid of MobileNet-VGG19 are crucial for gauging the effectiveness of the models. The evaluation measures provide an alternative perspective on the model’s advantages and disadvantages. There are several matrices used to quantify performance, including accuracy, recall (sensitivity), specificity, and F1-score. These evaluation matrices, expressed by Equations (1–4), can be calculated from the confusion matrix.


(1) $$\mathrm{Accuracy}=\frac{TP+TN}{FP+FN+TP+TN}\times 100$$

(2) $$\mathrm{Recall}=\mathrm{Sensitivity}=\frac{TP\;}{TP+FP}\;\times 100\%$$

(3) $$\mathrm{Specificity}=\frac{TN}{TN+FN}\times 100\%$$

(4) $$F1-\mathrm{score}=2*\frac{\mathrm{precision}\times \mathrm{Recall}\;}{\mathrm{precision}+\mathrm{Recall}}\;\times 100\%$$

where *TP, TN, FP*, and *FN* stand for true positives, true negatives, false positives, and false negatives, respectively.

## 4 Results

This section focuses on the gained testing results of each model for spotting ASD using eye-tracking characteristics. The testing process included evaluation of four separate deep learning models: MobileNet, VGG19, DenseNet169, and a combination of VGG19 and MobileNet called the hybrid model.

### 4.1 Models’ configuration

The efficacy of the advanced deep learning algorithm was evaluated in a specific environment to identify ASD using an eye-tracking method. Table 5 presents the environment of the DL models.

**Table 5:** Environment of the proposed DL.

GPU	GPU T4 Χ 2 Kaggle
Memory	4GB
Language	Python
TensorFlow	
Keras	
Panda	

### 4.2 Splitting dataset

The dataset was segregated into three subsets: training, testing, and validation. Table 6 displays the specific division that was employed in the proposed method for diagnosing ASD.

**Table 6:** Dataset.

Training set	77.78%
Validation set	22.22%
Testing set	10%

### 4.3 The test classification results of the MobileNet model

The MobileNet model demonstrated outstanding performance in all parameters, attaining perfect precision, recall, and F1-score for both ASD and non-ASD classes. This indicates that the model accurately categorized all cases of ASD and non-ASD without any incorrect positive or negative predictions, resulting in a remarkable overall accuracy of 100%. Table 7 presents the testing classification results of MobileNet.

**Table 7:** Testing classification results of the MobileNet model.

Class	Precision (%)	Recall (%)	F1-score (%)	Support (%)	Accuracy (%)
Non ASD	100	100	100	33	100
ASD	100	100	100	22	
Macro average	100	100	100	55	

**Figure 7:**
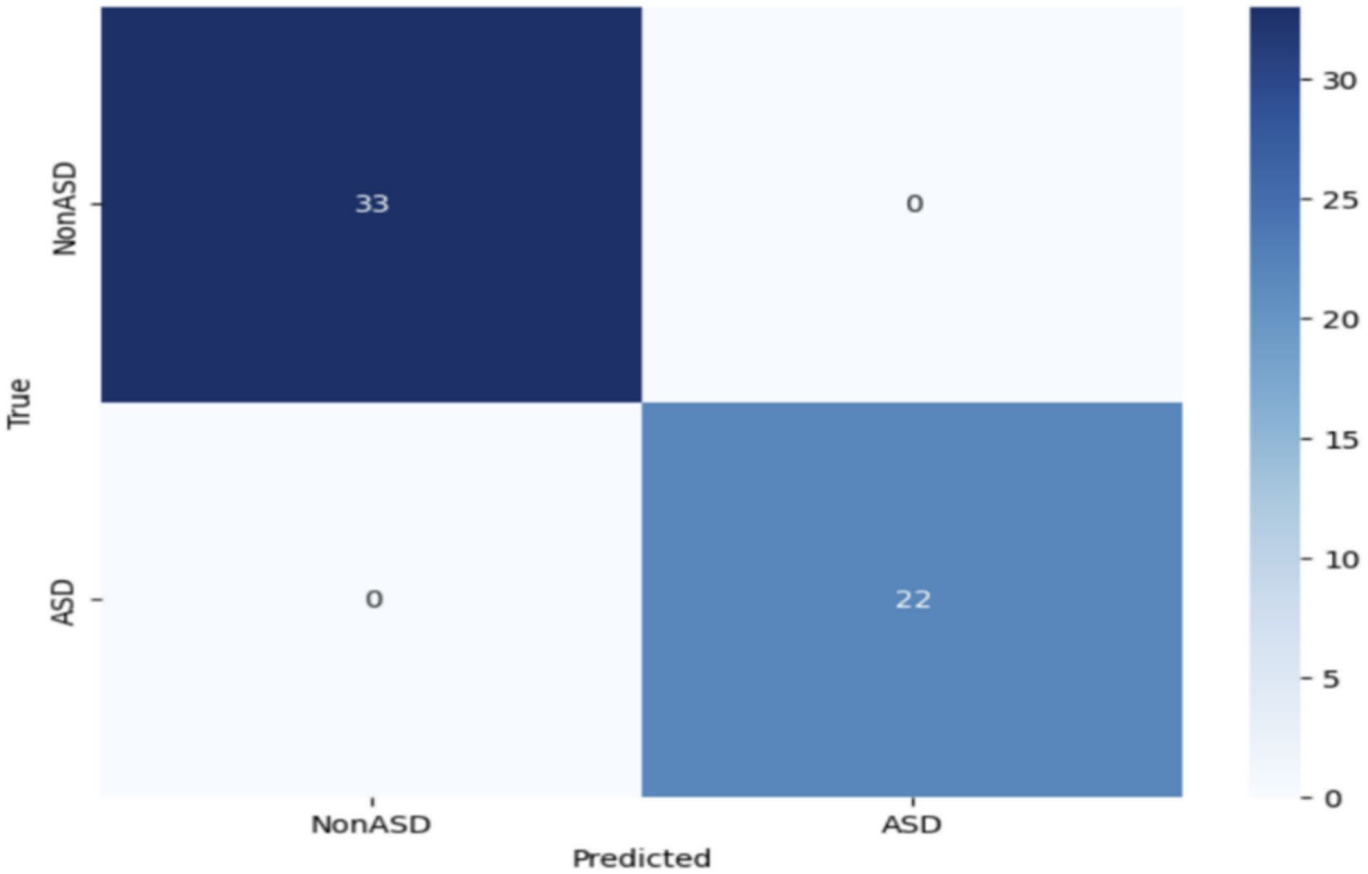
Confusion matrix of the MobileNet model.

The impressive performance of MobileNet underscores its efficacy in accurately recognizing instances of ASD through the utilization of eye-tracking characteristics. Figure 7 depicts the confusion matrix, which reveals that 33 images were correctly identified as true negatives (TN), 22 images were correctly classified as true positives (TP), and there were no instances of false positives (FP) or false negatives (FN). Based on the empirical data, it has been determined that the MobileNet model obtained a high level of accuracy.

**Figure 8:**
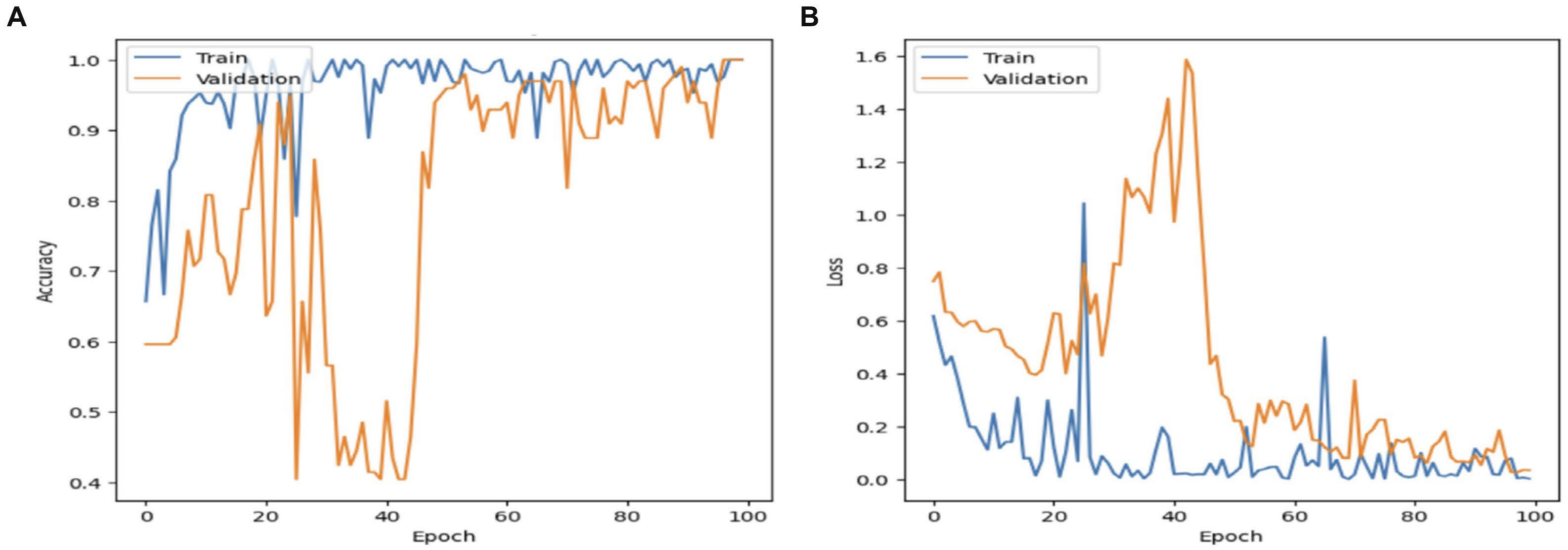
The MobileNet model: **(A)** Accuracy; **(B)** Loss.

Figure 8 displays the performance of the MobileNet model. The model’s accuracy exhibited a progressive increase in validation performance, starting at 50% and reaching 100%. In contrast, the accuracy in training performance had a smoothing effect, starting at 65% and also reaching 100%. The decline in the MobileNet starting and validation performance has resulted in a fall of 1.6% to reach 0.0. This confirms that the MobileNet model has achieved a high percentage score.

### 4.4 Testing results of the VGG19 model

This subsection introduces the testing classification results gained by the VGG19 model which achieved an accuracy of 87%, its recall, precision and F1-score for the ASD class were pointedly lower than those for the non-ASD class. This suggests that although the model demonstrated good performance in appropriately categorizing individuals without ASD, it encountered difficulties in correctly identifying individuals with ASD, resulting in a greater incidence of false negatives. Table 8 summarizes and presents the testing results of VGG19 model.

**Table 8:** Testing results of the VGG19 model.

Class	Precision (%)	Recall (%)	F1-score (%)	Support (%)	Accuracy (%)
Non ASD	82	100	90	33	87
ASD	100	68	81	22	
Macro average	91	84	86	55	

**Figure 9:**
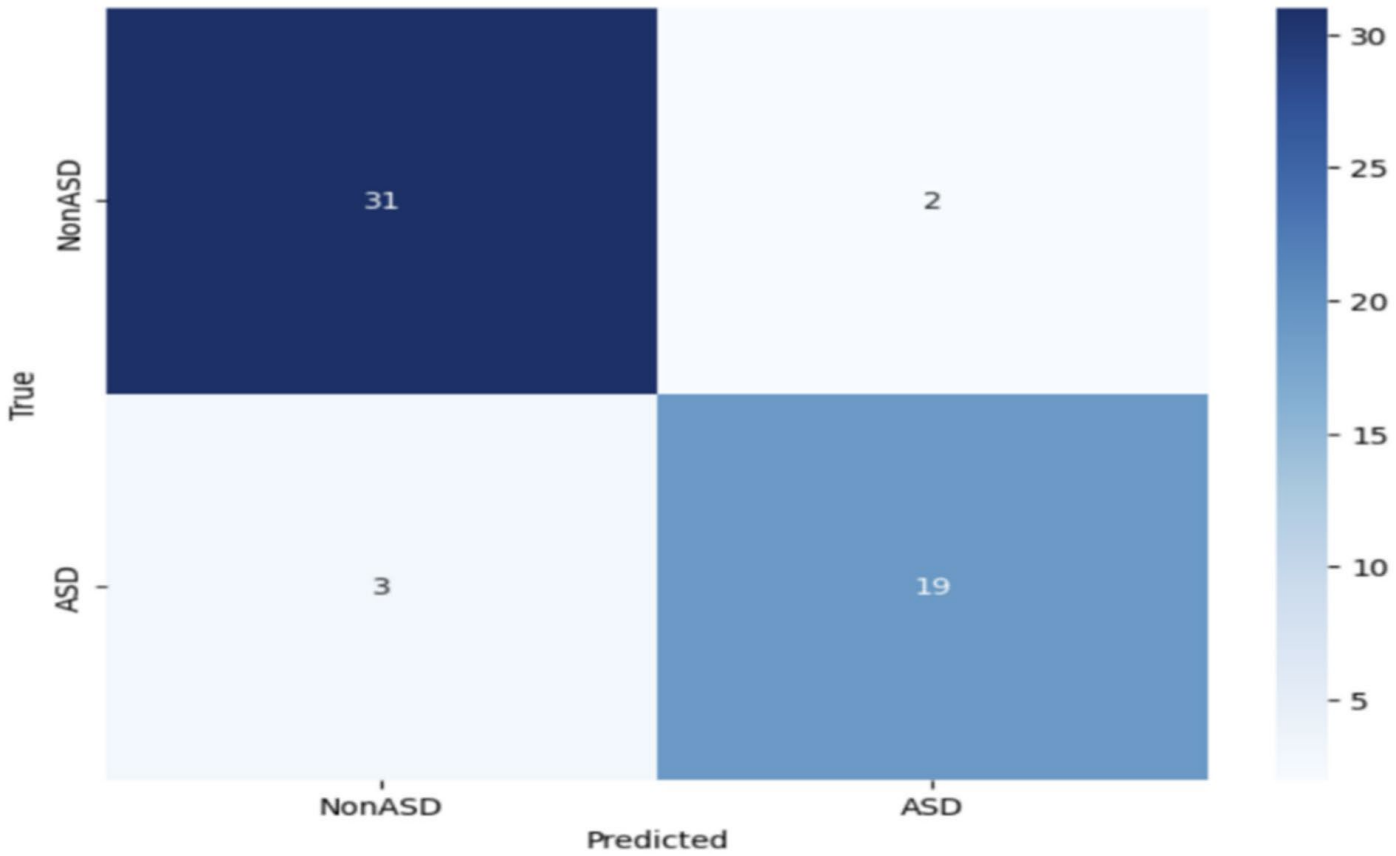
Confusion matrix of the VGG19 model.

Further modification or improvement of the VGG19 design may be required to enhance its effectiveness in diagnosing ASD. Figure 9 depicts the confusion matrix of the VGG19 model used to categorize Autism Spectrum Disorder (ASD) using an eye-tracking method. The VGG19 model correctly identified 31 images as true negatives (TN) and 19 images as true positives (TP). However, it misclassified 3 images and incorrectly classified 2 images as false negatives (FN).

**Figure 10:**
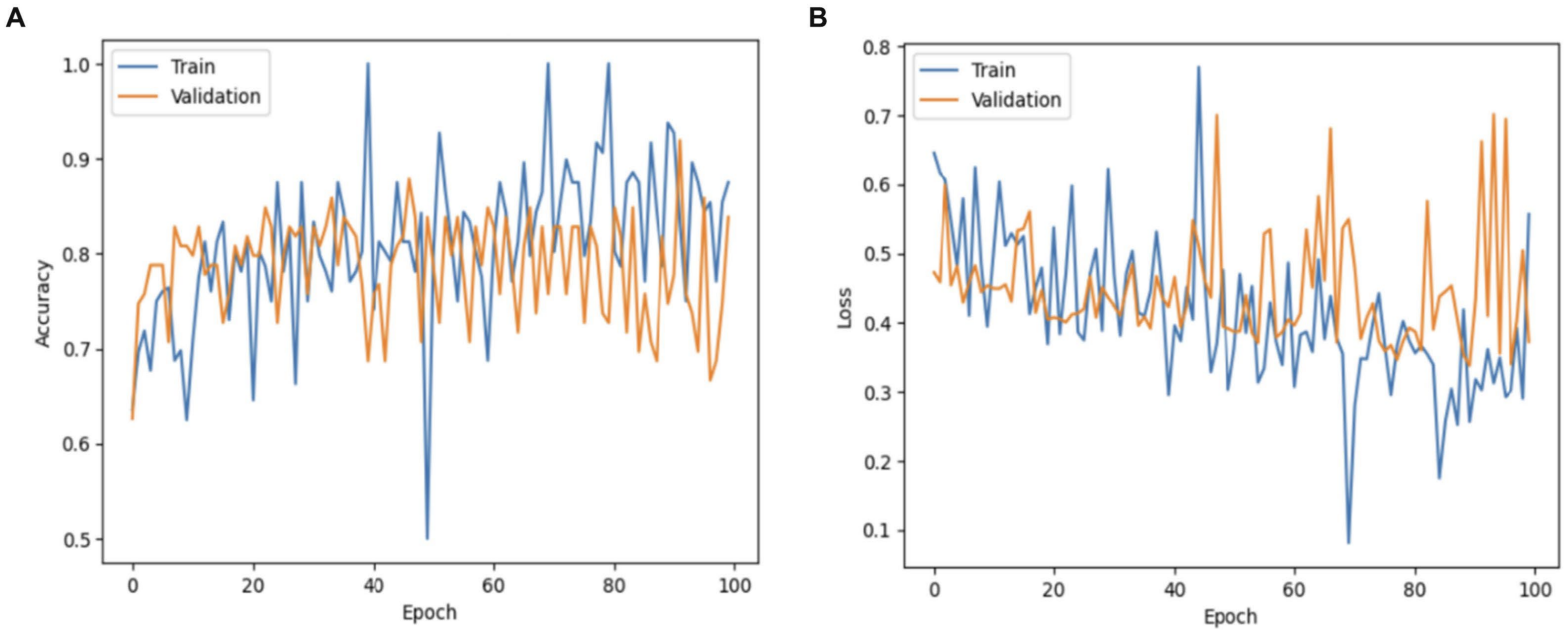
The VGG19 model: **(A)** Accuracy; **(B)** Loss.

Figure 10 illustrates the process of validating and training the VGG19 model. The VGG19 model achieved a validation accuracy of 87%. The VGG19 model attained an accuracy rate of 89% in diagnosing Autism Spectrum Disorder (ASD) using the eye-tracking dataset during training. The loss of the VGG19 model decreased to 0.3.

### 4.5 Testing classification results of the hybrid VGG19-MobileNet model

The hybrid VGG19-MobileNet model exhibited strong performance, with a 91% accuracy with well-balanced precision, recall, and F1-score for both ASD and non-ASD categories. The hybrid model successfully utilized the advantageous qualities of both VGG19 and MobileNet architectures, leading to enhanced classification performance. Table 9 presents the testing classification results obtained by the hybrid VGG19-MobileNet model.

**Table 9:** Testing results of the hybrid model.

Class	Precision (%)	Recall (%)	F1-score (%)	Support (%)	Accuracy (%)
Non ASD	91	94	93	33	91
ASD	90	86	88	22	
Macro average	91	90	90	55	

**Figure 11:**
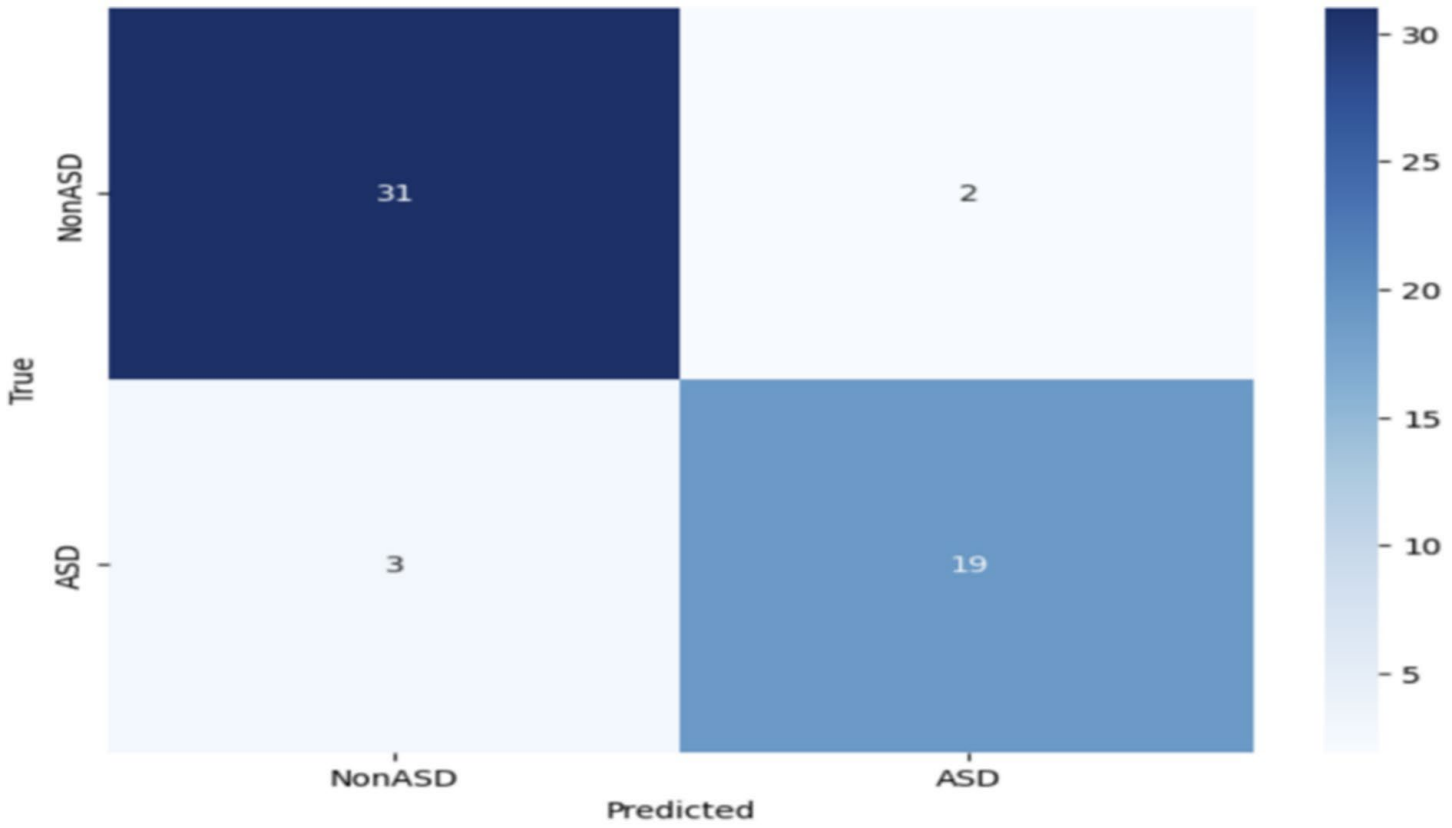
Confusion matrix of the hybrid model.

The model’s ability to accurately differentiate between cases of ASD and non-ASD highlights its potential utility in clinical settings for diagnosing ASD based on eye-tracking features. Figure 11 presents the confusion matrix of the hybrid VGG19-MobileNet model. In this hybrid model, 31 images were accurately labeled as TD and 19 images were accurately classified as ASD (autism spectrum disorder). The hybrid model correctly classifies 3 images as FP and incorrectly classifies 2 images as FN.

**Figure 12:**
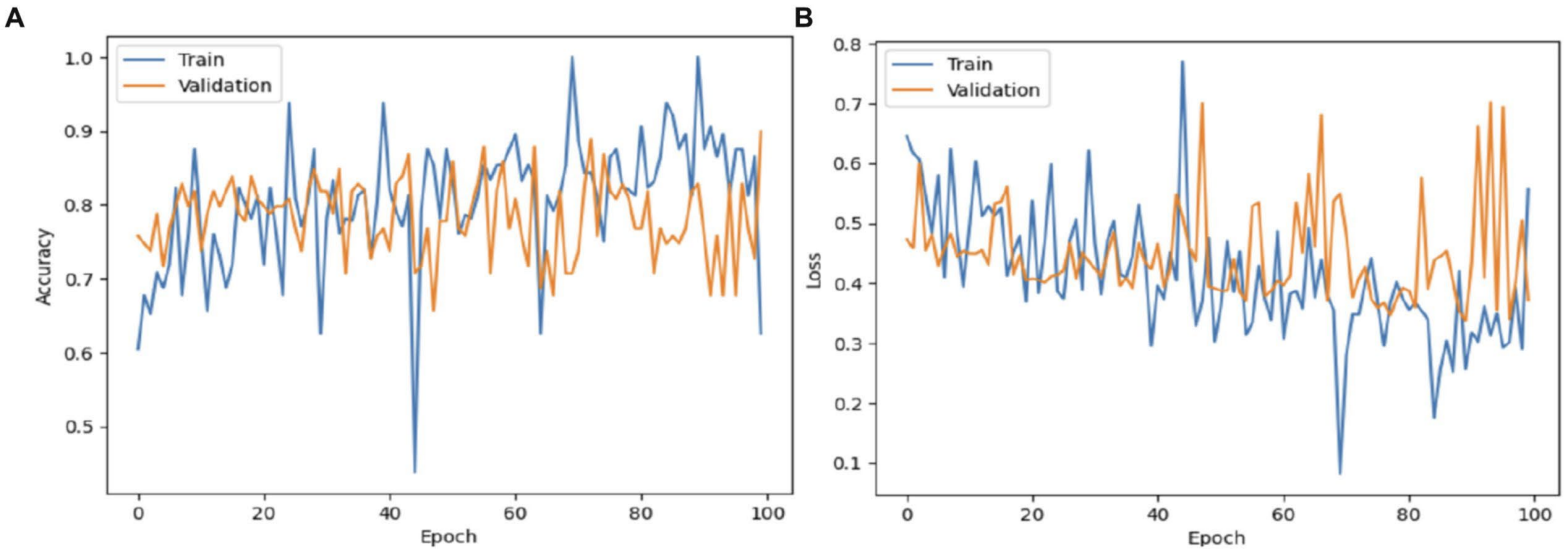
The Hybrid model: **(A)** Accuracy; **(B)** Loss.

The results performance of the VGG19-MobileNet model is depicted in Figure 12. The VGG19-MobileNet model obtained a validation accuracy of 91% and a training accuracy of 92%. The hybrid model had a reduction from 0.6 to 0.4.

### 4.6 Testing results of the DenseNet169 model

The DenseNet169 model attained an accuracy of 78%, exhibiting superior precision, recall, and F1-score for the non-ASD class in comparison to the ASD class. This indicates that although the model performed well in accurately categorizing those without ASD, its ability to identify individuals with ASD was comparatively less effective. Table 10 summarizes the testing classification results of the DenseNet169 model.

**Table 10:** Testing results of the DenseNet169 model.

Class	Precision (%)	Recall (%)	F1-score (%)	Support (%)	Accuracy (%)
Non ASD	74	97	84	33	78
ASD	92	50	65	22	
Macro average	83	73	74	55	

The elevated rate of false negatives in ASD cases highlights possible opportunities for enhancing the model’s ability to detect ASD-related characteristics. In general, although all models demonstrated potential in detecting ASD, there is a need for more improvement and optimization of model structures to boost the accuracy and precision of ASD diagnosis using eye-tracking data.

## 5 Discussion

ASD is a neurodevelopmental condition marked by enduring difficulties in social interaction, communication, and restricted or repetitive behaviors. People with Autism Spectrum Disorder (ASD) can display a diverse array of symptoms and levels of functioning, resulting in significant variation within the spectrum. Eye-tracking technology is the technique of observing and documenting the movement of a person’s eyes in order to examine different aspects of visual attention, perception, and cognitive processing. Eye-tracking studies in individuals with ASD commonly examine gaze fixation patterns, saccades (quick eye movements), and pupil dilation to explore disparities in visual processing and social attention between individuals with ASD and those who are typically developing.

The experimental results presented in this study demonstrate the efficacy of several convolutional neural network (CNN) models in detecting and predicting Autism Spectrum Disorder (ASD) by utilizing eye-tracking features. The classification accuracy, precision, recall, and F1-score of each model offer valuable insights into their efficacy in detecting ASD cases using eye movement patterns.

The MobileNet model exhibited outstanding performance, attaining flawless precision, recall, and F1-score for both ASD and non-ASD categories. This indicates that MobileNet successfully diagnosed all cases of ASD and non-ASD, demonstrating its potential usefulness in diagnosing ASD using eye-tracking data.

Although the VGG19 model achieved an accuracy of 87%, its precision, recall, and F1-score for the ASD class were somewhat lower, suggesting a higher occurrence of false negatives. This implies that VGG19 might have difficulties in reliably detecting cases of ASD solely based on eye movement patterns.

The DenseNet169 model attained an accuracy of 78%, exhibiting superior precision, recall, and F1-score for the non-ASD class in comparison to the ASD class. This disparity suggests possible constraints in the model’s ability to detect ASD-related eye movement characteristics, resulting in an increased occurrence of incorrect negative diagnoses for individuals with ASD.

The hybrid VGG19-MobileNet model exhibited strong performance, with a 91% accuracy with well-balanced precision, recall, and F1-score for both ASD and non-ASD categories. This suggests that the hybrid model successfully utilized the advantages of both VGG19 and MobileNet architectures to enhance ASD identification using eye-tracking features.

**Figure 13:**
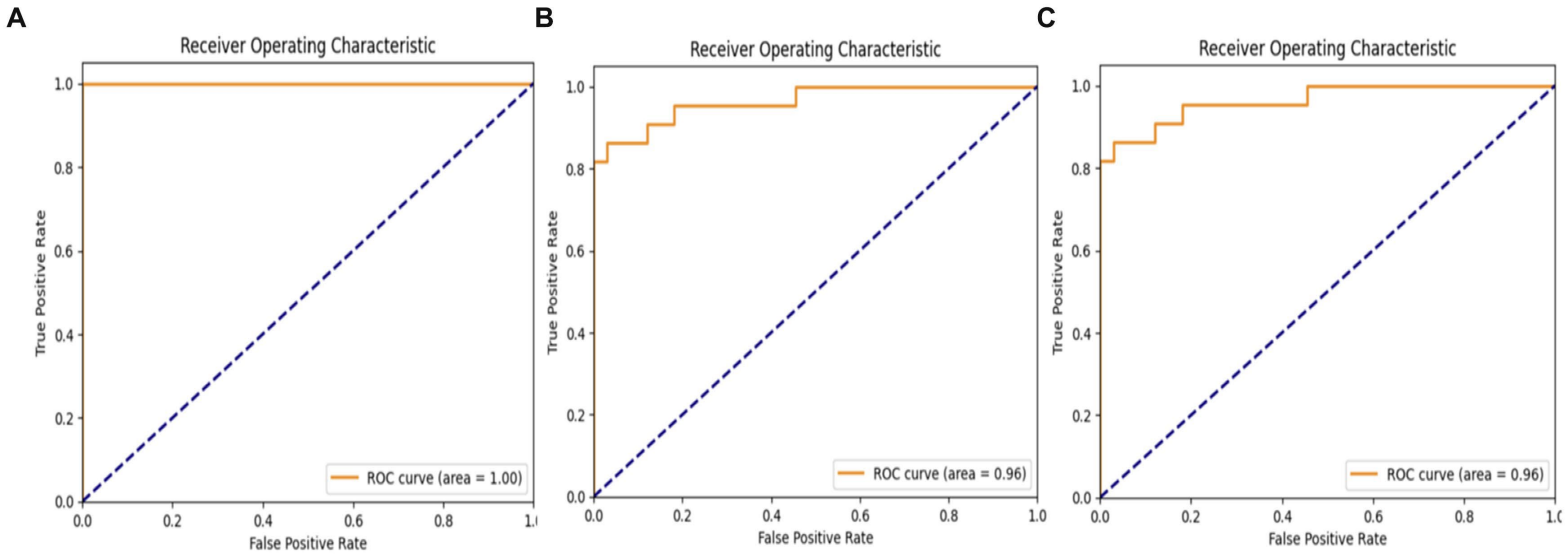
ROC of: **(A)** MobileNet model; **(B)** VGGA19 model; **(C)** Hybrid model.

Figure 13 displays the receiver operating characteristics (ROC) findings of the proposed deep learning (DL) model. The MobileNet model earned a high accuracy score of 100%, while both the VGG19 and hybrid models achieved the same accuracy score of 96%.

**Figure 14:**
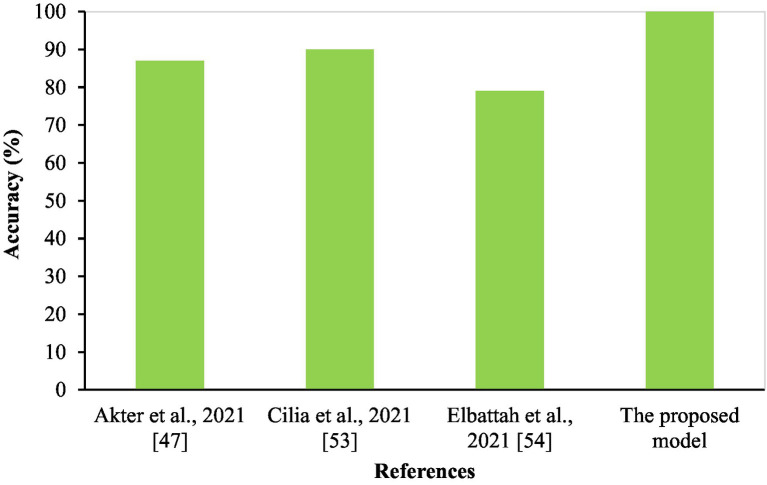
Accuracy of the proposed eye-tracking diagnostic system compared with other systems.

In summary, the experimental results highlight the capability of CNN models, specifically MobileNet and the hybrid VGG19-MobileNet model, to accurately detect ASD cases using eye-tracking data. However, additional study is required to optimize the design of models and increase their ability to detect patterns in eye movements associated to ASD. This will ultimately lead to better accuracy in diagnosing and treating ASD. The proposed system was compared to several current eye-tracking systems (46–48), as seen in Table 11 and Figure 14. Our enhanced MobileNet model achieved a perfect score of 100%, surpassing all other current systems.

**Table 11:** Results of the proposed eye-tracking diagnosis system compared with other systems.

Authors, years	Dataset	Approach	Accuracy %
Akter et al., 2021 (47)	Same	DT, SVM, LR, KNN, and MLP	Accuracy (87%), and AUC (79%)
Cilia et al., 2021 (53)	Same	CNN	90%
Elbattah et al., 2021 (54)	Same	Variational Autoencoder (VAE)	79%
This proposed model	Same	MobileNet	Accuracy (100%), and ROC (100%)

## 6 Conclusion

Eye tracking is a commonly used method for detecting ASD in both young children and adults. Research including eye tracking has revealed that individuals with autism have distinct gaze patterns compared to normally developing individuals. Various diagnostic procedures have been considered for the diagnosis of ASD, such as parent interviews, homogenous behavioral appraisals, and neurological examinations. Eye-tracking technology has gained significance for supporting the study and analysis of autism. This research presents a methodology that utilizes advanced deep learning algorithms, including MobileNet, VGG19, DenseNet169, and a hybrid of MobileNet-VGG19, to analyze and display the eye-tracking patterns of persons diagnosed with ASD. The study specifically focuses on children and adults in the initial phases of growth. The primary concept is to convert the movement patterns of the eye into a visual depiction, allowing for the use of image-based methods in activities connected to diagnosis. The visualizations generated are freely accessible as an image collection for use by other studies seeking to explore the capabilities of eye-tracking in the setting of Autism ASD. The collection consists of 547 images, with 328 images representing persons without ASD and 219 images representing those diagnosed with ASD. The MobileNet model scored high accuracy 100%, the proposed methodology was compared with different with existing ASD model, it is investigated that our model out performance.

An important avenue for future study is to expand the sample size by include a wider range of participants, including a greater number of persons with ASD and TD individuals. By increasing the size of the sample, researchers might potentially uncover additional patterns and subtleties in the data.

## Data availability statement

Publicly available datasets were analyzed in this study. This data can be found at: https://figshare.com/articles/dataset/Visualization_of_Eye-Tracking_Scanpaths_in_Autism_Spectrum_Disorder_Image_Dataset/7073087.

## Author contributions

NA: Conceptualization, Data curation, Formal analysis, Funding acquisition, Investigation, Methodology, Project administration, Resources, Supervision, Validation, Writing – original draft. MA-A: Conceptualization, Data curation, Formal analysis, Investigation, Methodology, Resources, Software, Validation, Writing – original draft. MA-Y: Visualization, Writing – original draft, Writing – review & editing. NF: Conceptualization, Formal analysis, Writing – review & editing. ZK: Conceptualization, Formal analysis, Writing – review & editing.
